# Two new cave-dwelling species of the millipede genus *Paracortina* Wang & Zhang, 1993 from southern China (Diplopoda, Callipodida, Paracortinidae)

**DOI:** 10.3897/zookeys.517.9949

**Published:** 2015-08-13

**Authors:** Weixin Liu, Mingyi Tian

**Affiliations:** 1 Department of Entomology, College of Agriculture, South China Agricultural University, 483 Wushanlu, Guangzhou, 510640, China

**Keywords:** *Paracortina*, new species, taxonomy, cave-dwelling, China

## Abstract

Two new species of the millipede genus *Paracortina* Wang & Zhang, 1993 are described. Both are presumed troglophiles: *Paracortina zhangi* **sp. n.** from a cave in Ceheng County, southwestern Guizhou Province and *Paracortina yinae* **sp. n.** from a cave in Longlin County, western Guangxi Zhuang Autonomous Region. A distribution map and a key to all 12 *Paracortina* species are also provided.

## Introduction

The family Paracortinidae was first established to comprise three genera: *Paracortina* Wang & Zhang, 1993, *Relictus* Wang & Zhang, 1993, *Altum* Wang & Zhang, 1993 (Wang and Zhang 1993). A fourth genus, *Angulifemur* Zhang, 1997, was added soon thereafter (Zhang 1997). All these genera are distributed in southern China only Yunnan and Sichuan provinces, as well as Tibet (Xizang Automomous Region).

The family has since been reviewed and shown to have *Relictus* and *Altum* as junior synonyms of *Paracortina* (Stoev and Geoffroy 2004). The genus *Scotopetalum* Shear, 2000, which only included a single species from Vietnam, has been synonymized with *Paracortina* (Stoev and Geoffroy 2004). Although *Angulifemur* Zhang, 1997, with two species still remains treated as an independent genus, it is likely to also represent only a junior synonym of *Paracortina* (Stoev et al. 2008). In 2004, the first troglomorphic species *Paracortina wangi* Stoev was described from south Yunnan, but it was subsequently synonymized with *Angulifemur unidigitis* Zhang, 1997 (Stoev et al. 2008).

As a result, *Angulifemur* consists of two species only from Mengzi, Yunnan of China. *Paracortina* is the largest genus in the family Paracortinidae including ten species and ranging from Tibet, Sichuan and Yunnan in southwestern China in the north of its distribution to Thanh Hoa and Hoa Binh provinces of northern Vietnam in the south (Stoev et al. 2008, Stoev and Enghoff 2011).

*Angulifemur tridigitis* Zhang, 1997, from Mengzi City, Yunnan, China.

*Angulifemur unidigitis* Zhang, 1997, from Mengzi City, Yunnan, China.

*Paracortina carinata* Wang & Zhang, 1993, from Shangrila County (=Zhongdian County), Yunnan, China.

*Paracortina chinensis* Stoev & Geoffroy, 2004, from Zhenxiong County, Yunnan, China.

*Paracortina leptoclada* Wang & Zhang, 1993, from Shangrila County, Yunnan, China.

*Paracortina multisegmentata* Stoev & Geoffroy, 2004, from Ngoc-Lac and Loc Thinh, Thanh Hoa, Vietnam.

*Paracortina serrata* Wang & Zhang, 1993, from Deqin County, Yunnan, China.

*Paracortina stimula* Wang & Zhang, 1993, from Shangrila County, Yunnan, China.

*Paracortina thallina* Wang & Zhang, 1993, from Batang County, Sichuan, and Shangrila County, Yunnan, China.

*Paracortina viriosa* Wang & Zhang, 1993, from Shangrila County, Yunnan, and Mangkang County, Tibet, China.

*Paracortina voluta* Wang & Zhang, 1993, from Yajiang County, Sichuan, China.

*Paracortina warreni* Shear, 2000, from Hong Mat, Hoa Binh, Vietnam.

Of these, four species have been considered as troglophiles: *Angulifemur tridigitis*, *Angulifemur unidigitis*, *Paracortina chinensis*, and *Paracortina warreni* (Stoev and Geoffroy 2004, Stoev et al. 2008). The present paper records two new paracortinid millipedes found in two caves, one in Guizhou Province, the other in Guangxi Zhuang Autonomous Region, both in southern China.

## Material and methods

All types are deposited in the zoological collection of the South China Agricultural University, Guangzhou, China (SCAU). All specimens used in this study were collected by hand in caves and preserved in 75% ethanol.

Observations and dissections were performed using a Leica DFC295 stereoscope. The line illustrations were executed with the help of a Leica MZ125 stereoscope and a *camera lucida* attached to the stereoscope. The photographs were taken with a Canon EOS 40D camera, further processed using Adobe Photoshop CS5 computer software. The distribution map was created using MapInfo Professional 12.0 software.

The terminology used in the text is after Wang and Zhang (1993) and Stoev (2004).

## Taxonomic treatment

### Paracortina zhangi

Taxon classificationAnimaliaCallipodidaParacortinidae

Liu & Tian sp. n.

http://zoobank.org/A1DB7B9A-F062-46C0-BAB4-5EA2CDC815FA

Figs 1–6 , 7 , 8–10 , 11–14 , 15–18 , 19–22

#### Material examined.

Holotype: adult male (SCAU), China, Guizhou, Qianxinan Zizhizhou, Ceheng County, Rongdu Village, Cave Qiaoxia Dong, 24°03.008N, 105°43.147E, 964 m, 26.XII.2012, leg. Mingyi Tian, Weixin Liu, Feifei Sun & Haomin Yin. Paratypes. 1 male, 3 females, 10 juveniles (SCAU), same locality, together with holotype.

#### Description.

**Figures 1–6.**
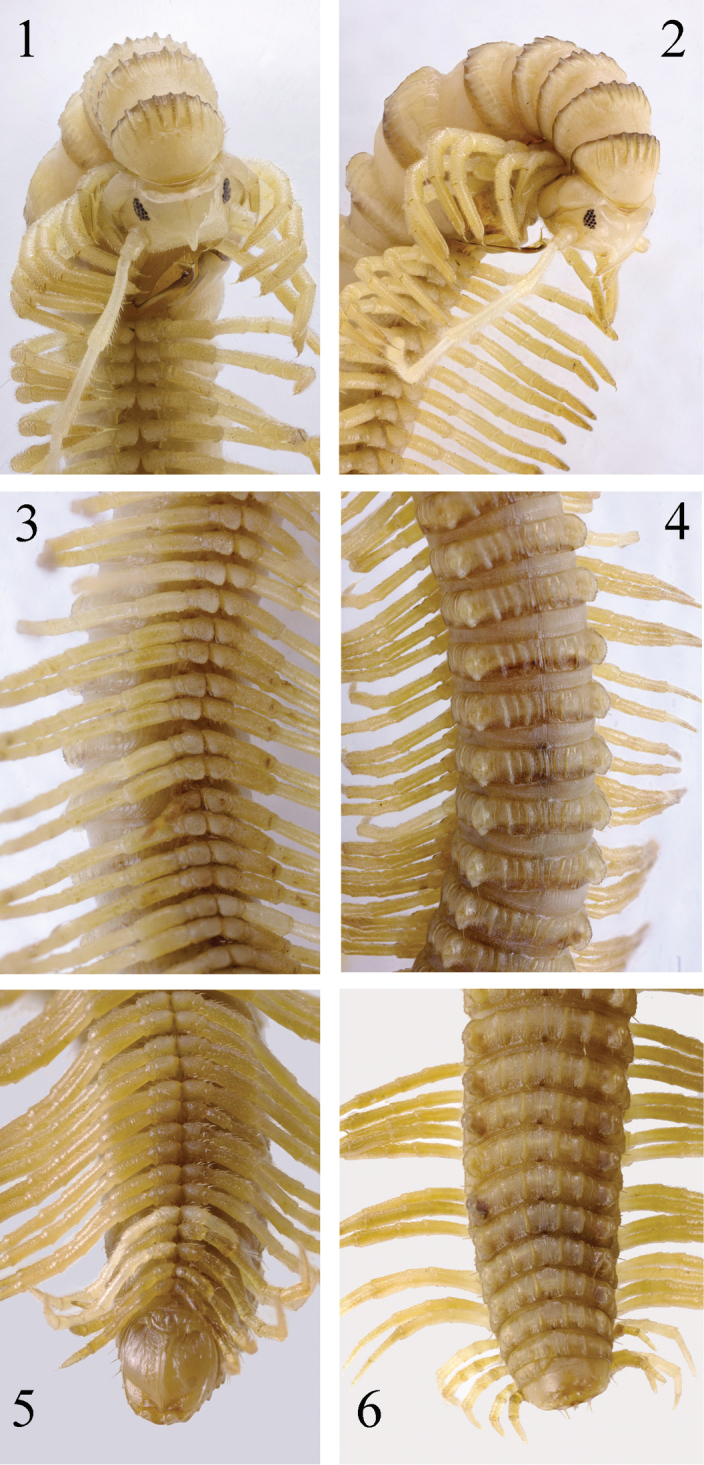
*Paracortina zhangi* sp. n. holotype **1** anterior body, subventral view **2** anterior body, sublateral view **3** midbody, ventral view **4** midbody, dorsal view **5** posterior body, ventral view **6** posterior body, dorsal view.

**Figure 7.**
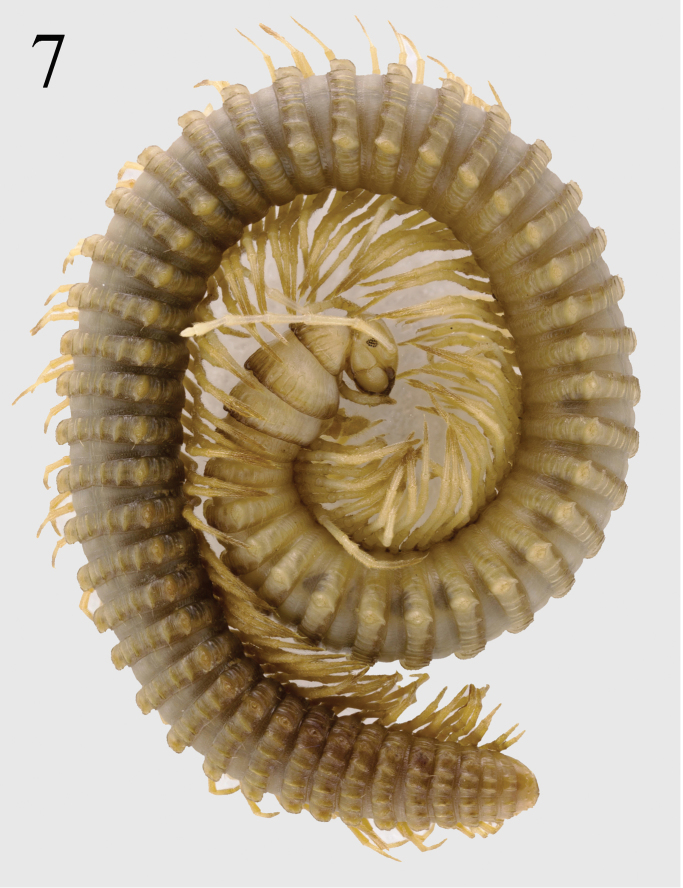
*Paracortina zhangi* sp. n. female paratype.

Length of adults of both sexes 46–55 mm, width of midbody segments 2.6–3.0 mm, body with 55–58 pleurotergites + telson. Holotype 46 mm long, 2.6 mm wide on midbody segment, maximum width on 6^th^ pleurotergite 3.5 mm, body with 57 pleurotergites + telson. Body coloration light yellow-brownish, anterior part of body slightly lighter. Metazonae slightly darker than prozonae, posterior margin of pleurotergites brownish to dark brown, more infuscate on anterior pleurotergites (Figs 1–7). Head yellowish, epicranial suture distinct, with a large, median, beak-shaped process located between antennae in males, below it densely and finely setose and granulate (Figs 1–2). Edges of genae, posterior margin of the head, and bases of antennae marbled light brown-yellowish. Labrum dark brown. Ocellaria composed of ca. 16–23, dark grey ocelli arranged in four irregular longitudinal rows (Fig. 2). Tömösváry’s organs about 2–3 times larger than an ocellus, placed between ocellaria and base of antenna. Antennae light yellow, rather long and slender, extending behind posterior edge of pleurotergite 6 in males or pleurotergite 3(4) in females when stretched backwards; antennomere length ratios: 2=3>4=5>6>1>7, antennomeres 5 and 6 with a small distodorsal field of fine setae (Fig. 16). Legs light yellow to yellow-brownish, tarsi much darker (Figs 1–7).

Collum much narrower than head, pleurotergite 6 in males strongly enlarged (Fig. 2). Prozonae delicately alveolate-areolate; fine longitudinal striations in front of stricture between prozonae and metazonae. Crests on collum normal, extended forwards from about midlength (Figs 1–2). All crests on the metazonae well-developed, forming 5+5 primary crests, 5+5 secondary crests, and 12–18 lower crests down to ventral pleurotergal edge (Figs 4, 6). 3^rd^ primary crest strongly enlarged. Ozopores starting with pleurotergite 6, present until penultimate pleurotergite, placed on tip of 3^rd^ primary crest (Figs 2, 4, 7). 2+2 primary and 3+3 secondary crests between poriferous crests. Pleurotergal setae 5+5, located at edges of primary crests; setal pattern as in Table 1. Axial line rather distinct (Figs 4, 6).

**Table 1.** Chaetotaxy of *Paracortina zhangi* sp. n. and *Paracortina yinae* sp. n.

	**Anterior setae**	**Posterior setae**
Collum	5+5	-
Pleurotergites 2 to 4	5+5	-
Pleurotergite 5	d, a + a, d	e, c, b + b, c, e
Pleurotergite 6 to penultimate	-	5+5

**Figures 8–10.**
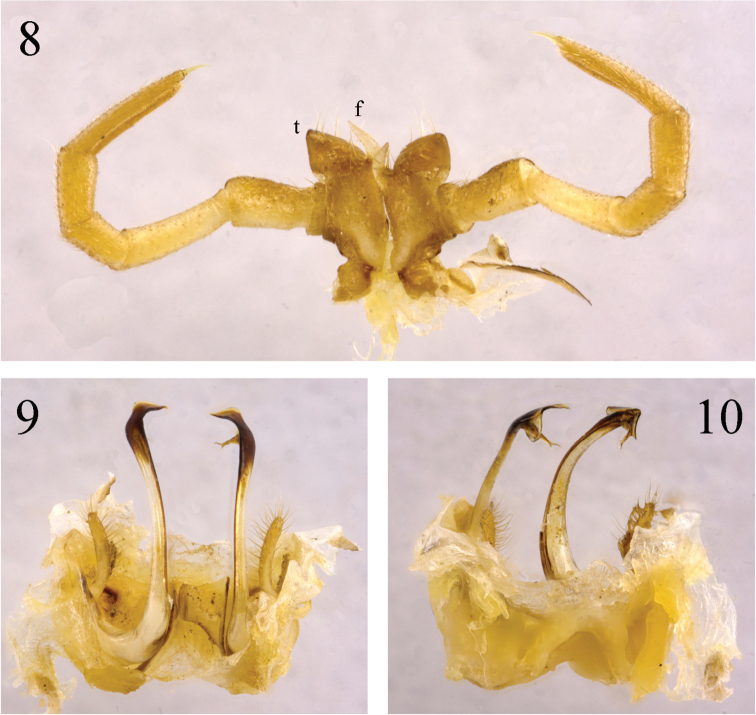
*Paracortina zhangi* sp. n. male paratype **8** leg-pair 7, anterior view **9** gonopods, dorsal view **10** gonopods, subventral view.

Male leg-pairs 1 and 2 much shorter, leg-pair 3 slightly shorter than following legs (Figs 1–2). Midbody legs about 4 (male) or 3 times (female) as long as pleurotergite height. Prefemora to a lesser extent, postfemora and tibiae more strongly, but still finely micropapillate ventrally (Fig. 15). Tarsi 1–3 only 1-segmented, from leg 4 to ultimate pair 2-segmented in both sexes; male tarsal pads visible from leg-pairs 3 to 23. All legs ending with a rather slender, long and curved claw (Figs 1, 2, 15). Coxal sacs present from leg 3 to 23. Male coxa 2 with a small anterior process and a posterior gonopore, the latter placed on a small cone. Coxa 7 with a long, subfalcate and apically pointed posterior (**f**), and a rather strong, pear-shaped anterior process (**t**) (Fig. 8); **f** in situ protruding between the gonopods. Coxae of remaining legs normal. Epiproct simple, with about 10 tuberculations in irregular rows (Fig. 6). Hypoproct tripartite, medial sclerite largest, subrectangular, bearing two paramedian macrosetae; each lateral sclerite with a single macroseta. Anal valves smooth, each divided into a small triangle and a large sclerite, both with a pair of macrosetae. Spinnerets comparatively thin and long, ending with a long macroseta (Fig. 5).

Gonopods (Figs 9–10, 17–18) yellow-brown to brown basally, seminal groove and telopodite black brown. Coxa with a large, curved, arch-shaped, anterior process (**a**) and a rather slender process (**b**), the latter a little more than half the length of telopodite. Prefemur with a large, clavate process (**c**) densely covered with long macrosetae. Femoroidal stem long, slender, rather clearly curved, directed cephalad. Telopodite’s terminal part twisted, with a large median (**l**), and a small lateral lobe (**h**). Solenomere (**s**) bifid, parasolenomere (**ps**) much shorter; seminal groove ending on the longer branch.

**Figures 11–14.**
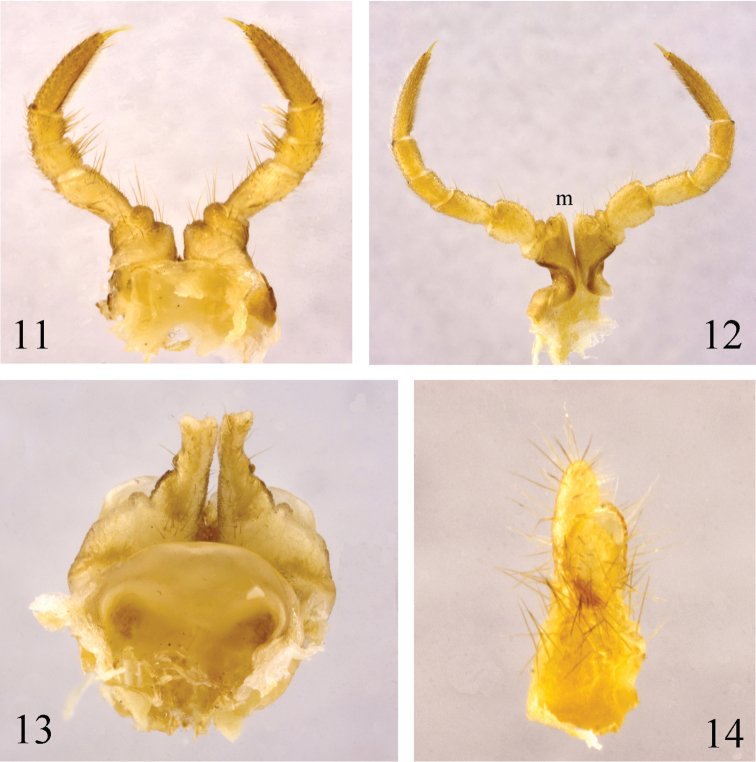
*Paracortina zhangi* sp. n. female paratype **11** leg-pair 1, anterior view **12** leg-pair 3, posterior view **13** leg-pair 2, anterior view **14** cyphopod, lateral view.

**Female.** Pleurotergites 2 and 3 greatly enlarged. Leg-pairs 1 and 3 with tarsal pads (Figs 11–12). Coxa 3 with a rather small process (**m**). Prefemora 3 and 4 relatively stout and clearly enlarged (Fig. 12). Leg-pair 2 (Fig. 13) rather strongly reduced down to a pairs of stout, apically shallowly biramous remnants in adult female (Fig. 7), normal in younger ones. Cyphopods small, densely setose, hidden in membranous sacs, divided laterally in the middle (Fig. 14). Coxae 7 normal, without processes.

**Figures 15–18.**
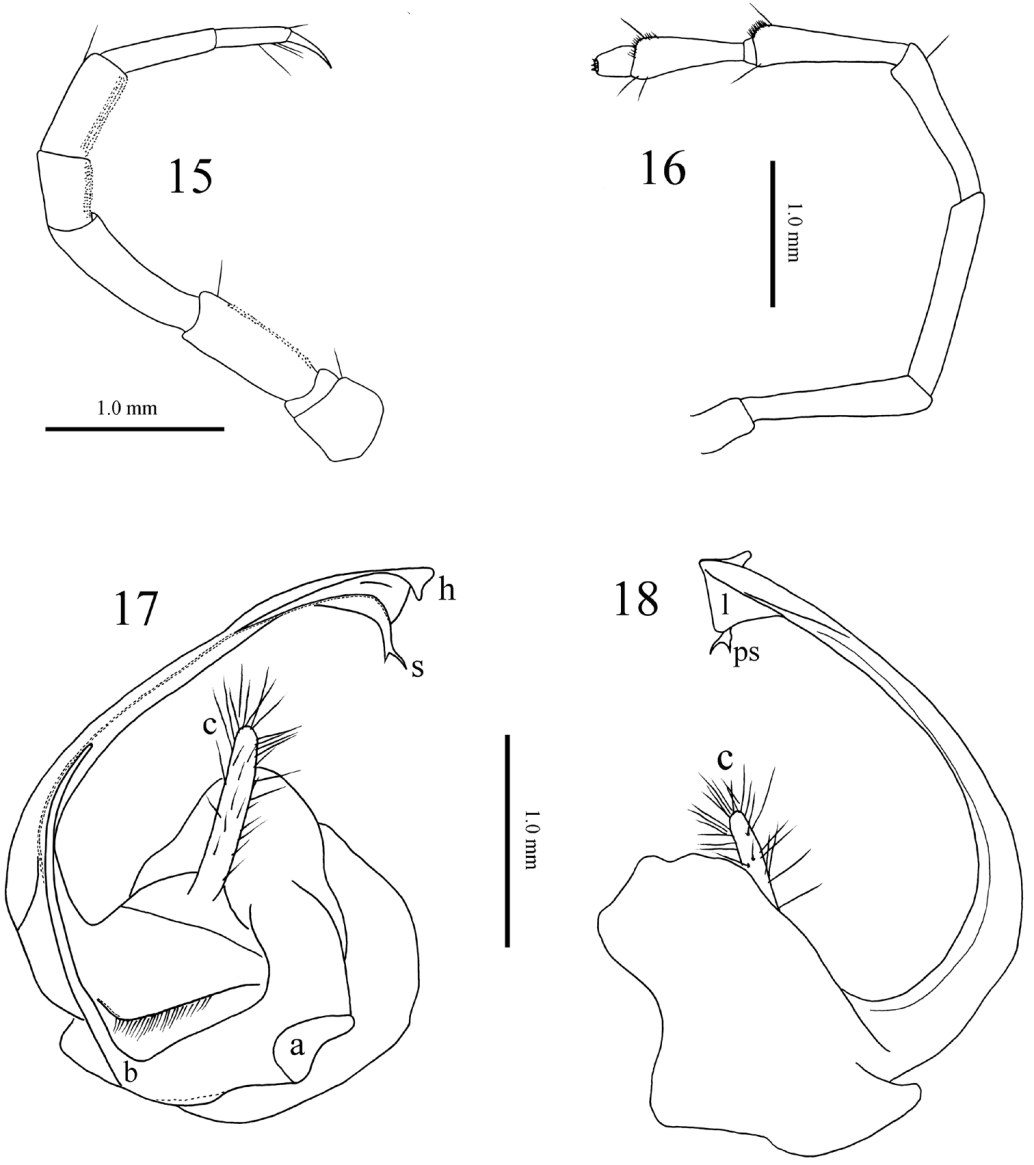
*Paracortina zhangi* sp. n. male paratype **15** midbody leg, anterior view **16** antenna, lateral view **17** right gonopod, mesal view **18** right gonopod, lateral view.

#### Etymology.

The species is dedicated to the memory of Mr. Chongzhou Zhang for his contribution to the systematics of Diplopoda in China.

#### Remarks.

This species is distinguished from its congeners by the presence of 5+5 primary crests, 5+5 secondary crests on the metazonae, two processes on coxa 7 in males, and in certain specific characters of the male gonopods, as well as in the small process on coxa 3, and reduced leg-pair 2 in females.

#### Distribution.

China: Guizhou (Fig. 46).

**Figures 19–22.**
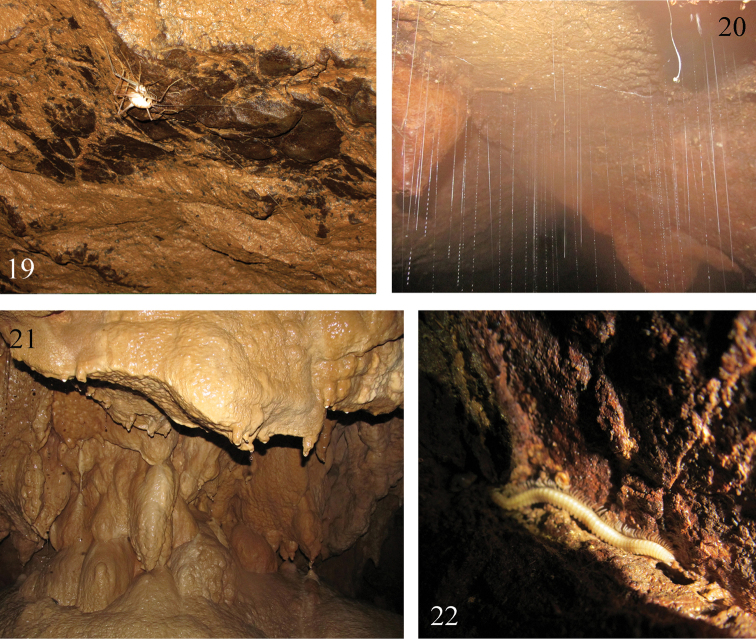
Cave Qiaoxia Dong **19–20** cave ceiling **21** cave deposits **22** *Paracortina zhangi* sp. n. walking on a rock.

The entrance of cave Qiaoxia Dong (Figs 19–22) is located under a bridge in the village of Rongdu. The cave is about 200 meters long, 10 meters wide and 5 meters high. It is rather polluted from sewage water and rubbish. Other animals living in this cave are the ubiquitous diplopod, *Oxidus gracilis* C. L. Koch, 1847 (Polydesmida, Paradoxosomatidae), some ground beetles, crickets (Fig. 19), glowworms (Fig. 20), and spiders, etc.

### Paracortina yinae

Taxon classificationAnimaliaCallipodidaParacortinidae

Liu & Tian sp. n.

http://zoobank.org/5F7E3FDD-A689-4F91-B2FE-5972754E0978

Figs 23–28 , 29 , 30–33 , 34–37 , 38–41 , 42–45

#### Material examined.

Holotype: adult male (SCAU), China, Guangxi, Baise City, Longlin County, Tianshengqiao Town, Yanchang Village, Cave I, 24.875732°N, 105.150143°E, 867 m, 12.VI.2014, leg. Mingyi Tian, Weixin Liu, Haomin Yin & Xiaozhu Luo. Paratypes. 2 males, 3 females, 2 juveniles (SCAU), same locality, together with holotype.

#### Description.

**Figures 23–28.**
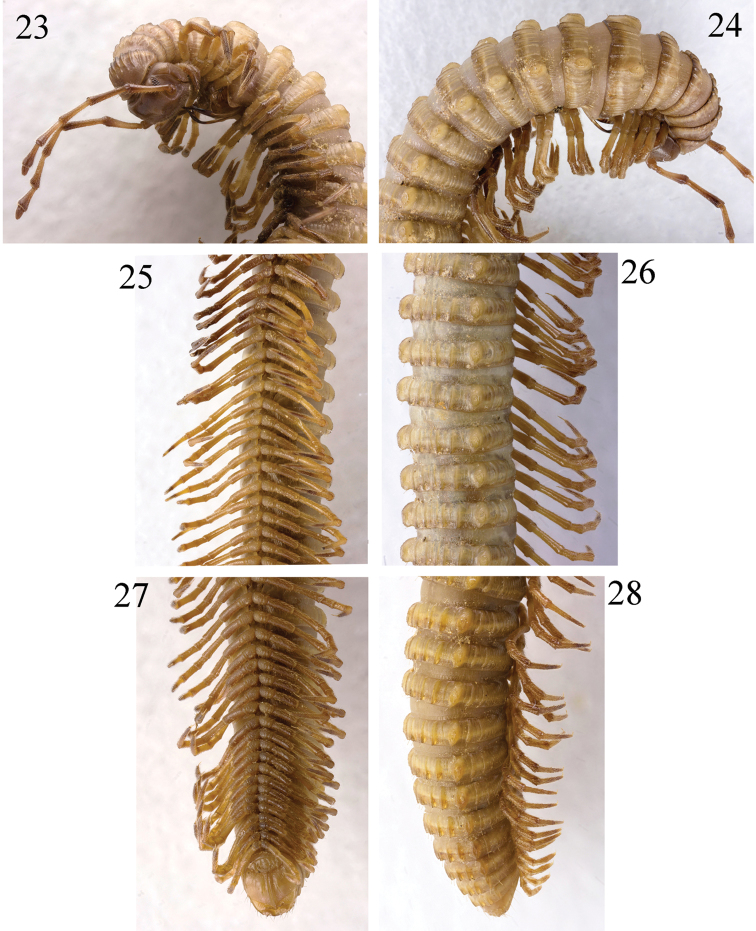
*Paracortina yinae* sp. n. holotype **23** anterior body, subventral view **24** anterior body, subdorsal view **25** midbody, ventral view **26** midbody, sublateral view **27** posterior body, ventral view **28** posterior body, lateral view.

**Figure 29.**
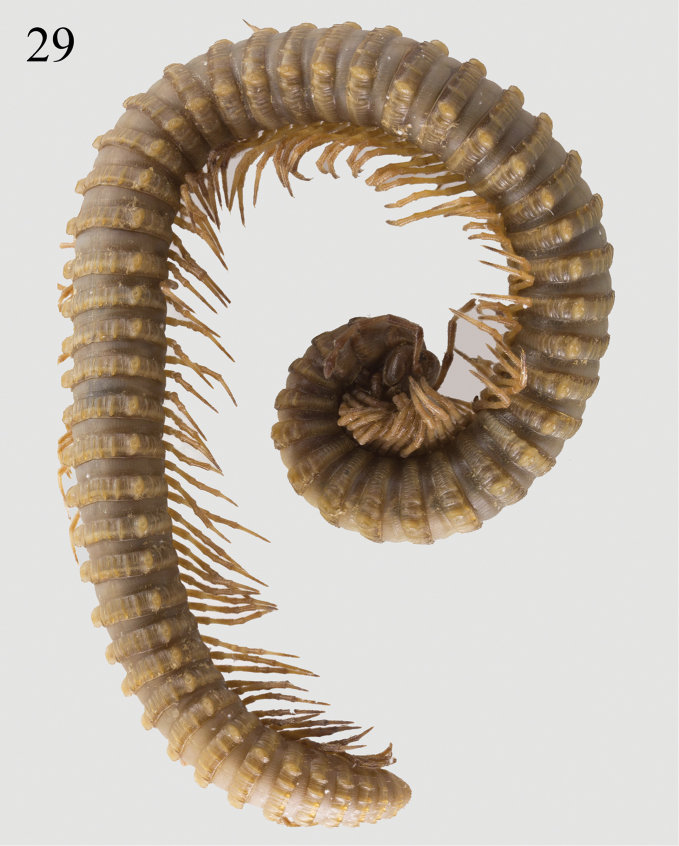
*Paracortina yinae* sp. n. female paratype.

Length of adult males 39–52 mm, of adult females 47–55 mm. Width of midbody segments in adult males 2.2–2.6 mm, in adult females 2.5–3.0 mm. Body with 53–61 pleurotergites + telson. Holotype 44.0 mm long, 2.5 mm wide on midbody segments, maximum width on 6^th^ pleurotergite 2.5 mm, body with 54 pleurotergites + telson. Body coloration yellow-brownish. Metazonae slightly darker than prozonae, posterior margin of pleurotergites dark brown to brownish, more evidently so on the anterior pleurotergites (Figs 23–29). Head brownish to chocolate brown, epicranial suture distinct, with a slightly smaller, median, beak-shaped process located between antennae in males, surface below the vertex and genae densely beset with brown granules and fine setae (Fig. 23). Genae, labrum, the edge between the dorsal and the frontal face of head marbled dark brown. Ocellaria composed of ca. 21–32, dark grey ocelli arranged in four irregular longitudinal rows. Tömösváry’s organs about 4 times larger than an ocellus, placed between ocellaria and base of antenna (Fig. 23). Antennae brownish, tip of each antennomere infuscate; very long, extending behind posterior edge of pleurotergite 6 (male) or 4 (female) when stretched backwards (Figs 23, 29), antennomere length ratios: 2=3>4=5>6>1>7, antennomeres 5 and 6 with a small distodorsal field of fine setae (Fig. 39). Legs yellow-brownish to dark brownish, distal parts of femora much darker.

Collum much narrower than head, with two paramedian spots covered with brown granules, pleurotergite 6 in males strongly enlarged. Prozonae delicately alveolate-areolate; fine longitudinal striations in front of stricture between pro- and metazonae. Two first primary crests on collum relatively large. All crests on the metazonae, ozopores site, pleurotergal setae, and axial line as in *Paracortina zhangi* sp. n. (Figs 24, 26, 28); setal pattern as in Table 1.

**Figures 30–33.**
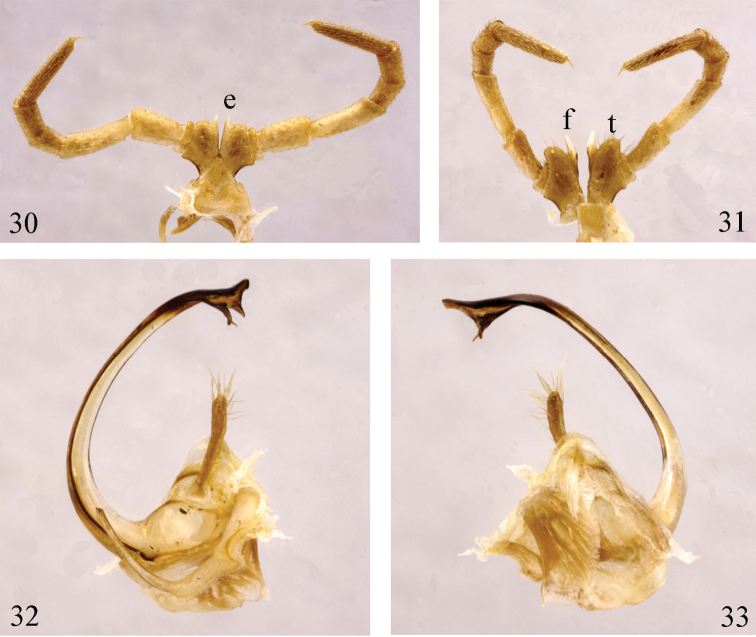
*Paracortina yinae* sp. n. male paratype **30** leg-pair 6, anterior view **31** leg-pair 7, anterior view **32** gonopods, mesal view **33** gonopods, lateral view.

Male leg-pairs 1 and 2 much shorter, leg-pair 3 slightly shorter than following legs (Fig. 23). Midbody legs about 4 (male) or 3 times (female) as long as pleurotergal height, only tibia finely micropapillate ventrally (Fig. 38). Tarsi 1–3 only 1-segmented, from 4 to ultimate pair 2-segmented; tarsal pads present from leg-pairs 3 to about 15. All legs ending with a rather slender, long and curved claw (Figs 1, 2, 15). Coxal sacs present from leg 3 to at least 25, but most abrased. Coxa 2 with a small anterior process and a posterior gonopore, the latter placed on a small cone. Coxa 6 with a small, pointed posterior process (**e**) (Fig. 30). Coxa 7 with a long, subfalcate and apically pointed posterior (**f**), and a very strong, rounded anterior process (**t**) (Fig. 31). Coxae of remaining legs normal. Epiproct simple, with 3+3 anterior and 8+8 posterior tuberculations in transverse rows. Hypoproct, anal valves and spinnerets as in *Paracortina zhangi* sp. n. (Fig. 27).

Gonopods (Figs 32–33, 40–41) yellow-brown to brown basally, seminal groove and telopodite black brown. Coxa with an anterior process (**a**) and a rather slender process (**b**), the latter about half the length of telopodite. Prefemur with a large, clavate process (**c**), densely covered with long macrosetae apically. Femoroidal stem long, slender, rather strongly curved, directed cephalad. Telopodite’s terminal part twisted, trifid, with a digitiform lateral (**h**), and a slender, apically pointed lobe (**t**). Solenomere (**s**) bifid, parasolenomere (**ps**) much shorter; seminal groove ending on the longer branch.

**Figures 34–37.**
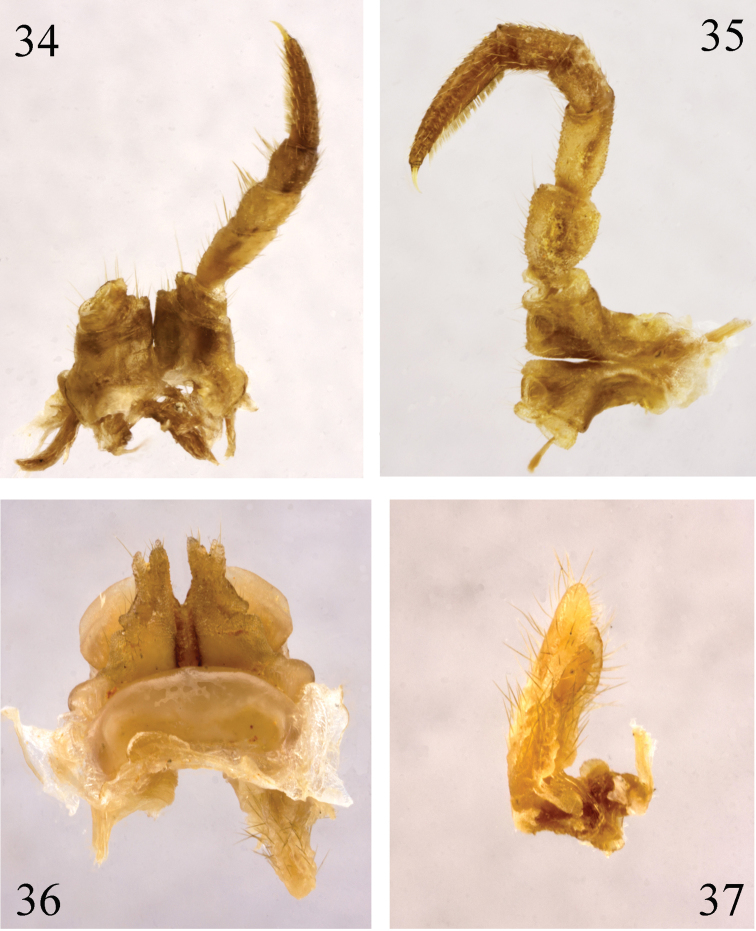
*Paracortina yinae* sp. n. female paratype **34** leg 1, anterior view **35** leg 3, posterior view **36** leg-pair 2 and cyphopod, anterior view **37** cyphopod, lateral view.

**Female.** A little larger than males, pleurotergites 2 and 3 strongly enlarged. Leg-pairs 1 and 3 with tarsal pads (Figs 34–35). Prefemur 3 evidently enlarged (Fig. 35). Leg-pair 2 (Fig. 36) rather strongly reduced down to a pairs of stout, apically deeply biramous remnants in adult females, normal in younger ones. Cyphopods small, densely setae, hidden in membranous sacs, partly extruded, laterally divided in the middle, the smaller part roundly concave apically (Figs 36–37). Coxae 6 and 7 normal, without processes.

**Figures 38–41.**
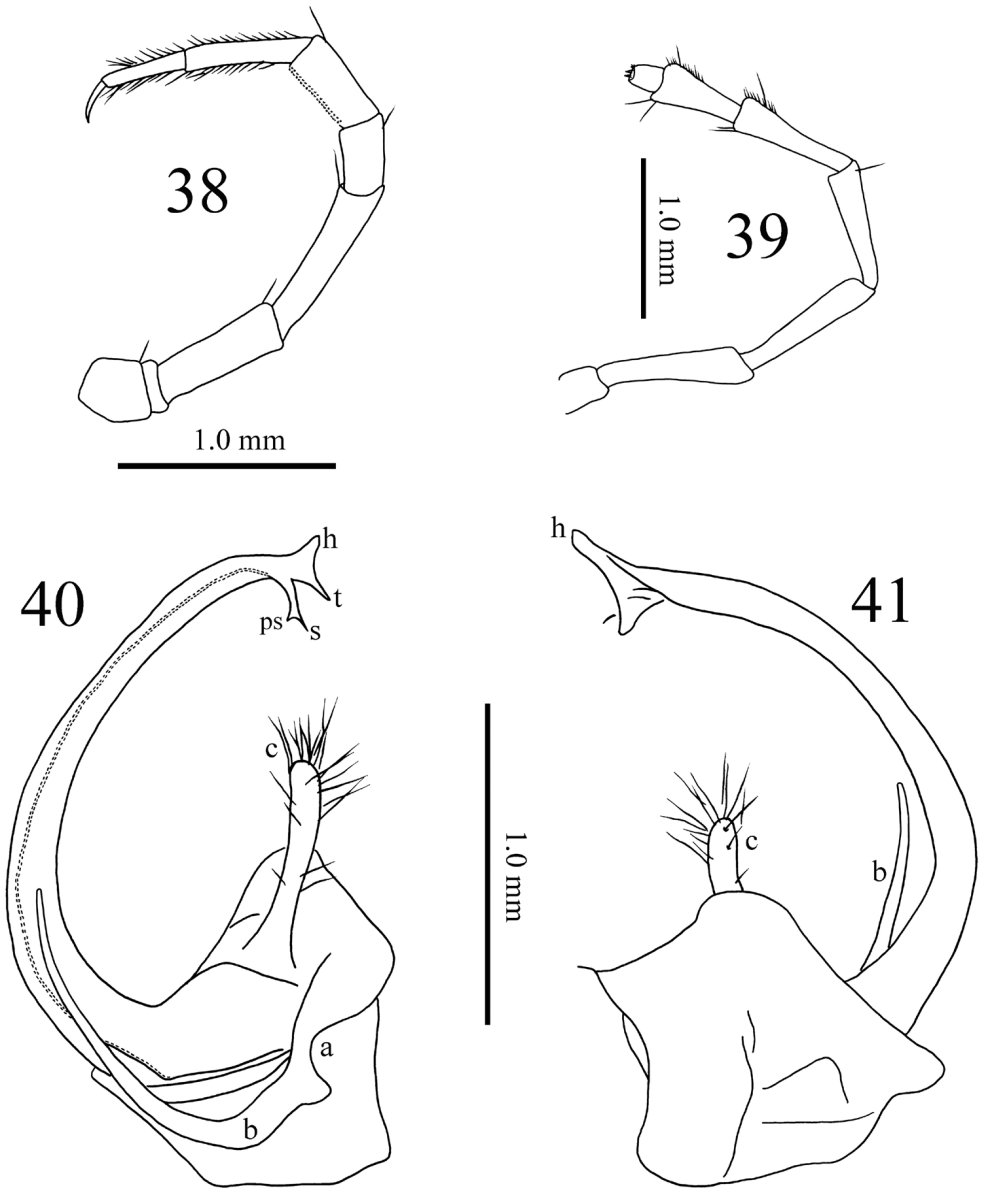
*Paracortina yinae* sp. n. male paratype **38** midbody leg, anterior view **39** antenna, lateral view **40** right gonopod, mesal view **41** right gonopod, lateral view.

#### Etymology.

The species is named in honour of Miss Haomin Yin, an active collector in our team.

#### Remarks.

The new species differs from its congeners by the presence of small, pointed, posterior processes (**e**) on coxae 6 and two pairs of processes on coxae 7 in males, as well as in certain specific charaters of the male gonopods and reduced leg-pair 2 in females.

#### Distribution.

China: Guangxi (Fig. 46).

**Figures 42–45.**
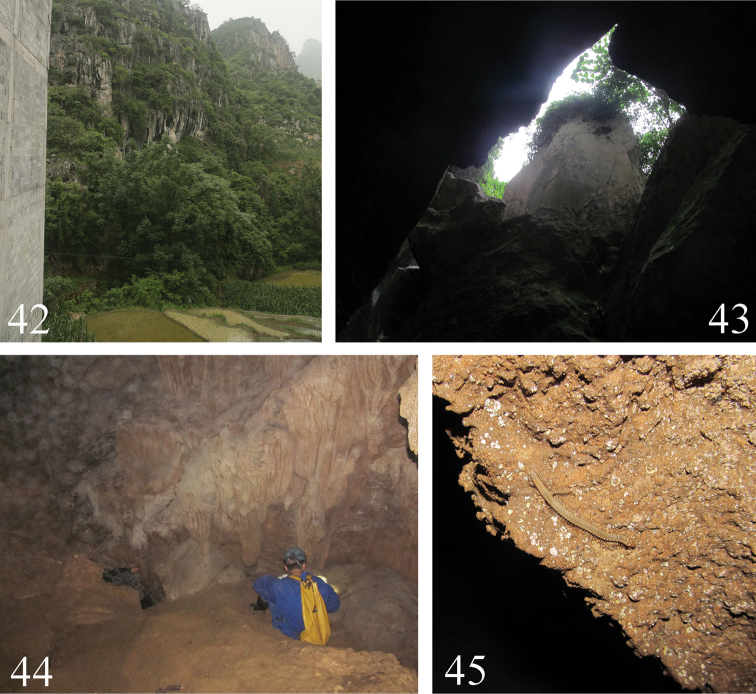
Cave I **42** location **43** entrance **44** cave walls **45** *Paracortina yinae* sp. n. walking on a rock.

Cave I (Figs 42–45) is situated at the foot of a karst mountain. It is a large cave, made up of a hall of several layers covered by numerous big rock boulders. We explored approximately 150 meters deep, but then had to withdraw because of a rushing underground river. Other animals also found in this cave are diplopods from the genera *Glyphiulus* (Spirostreptida, Cambalopsidae) and *Eutrichodesmus* (Polydesmida, Haplodesmidae), the blind trechine beetle *Satotrechus longlinensis* Deuve & Tian, 2011, and bats.

**Figure 46.**
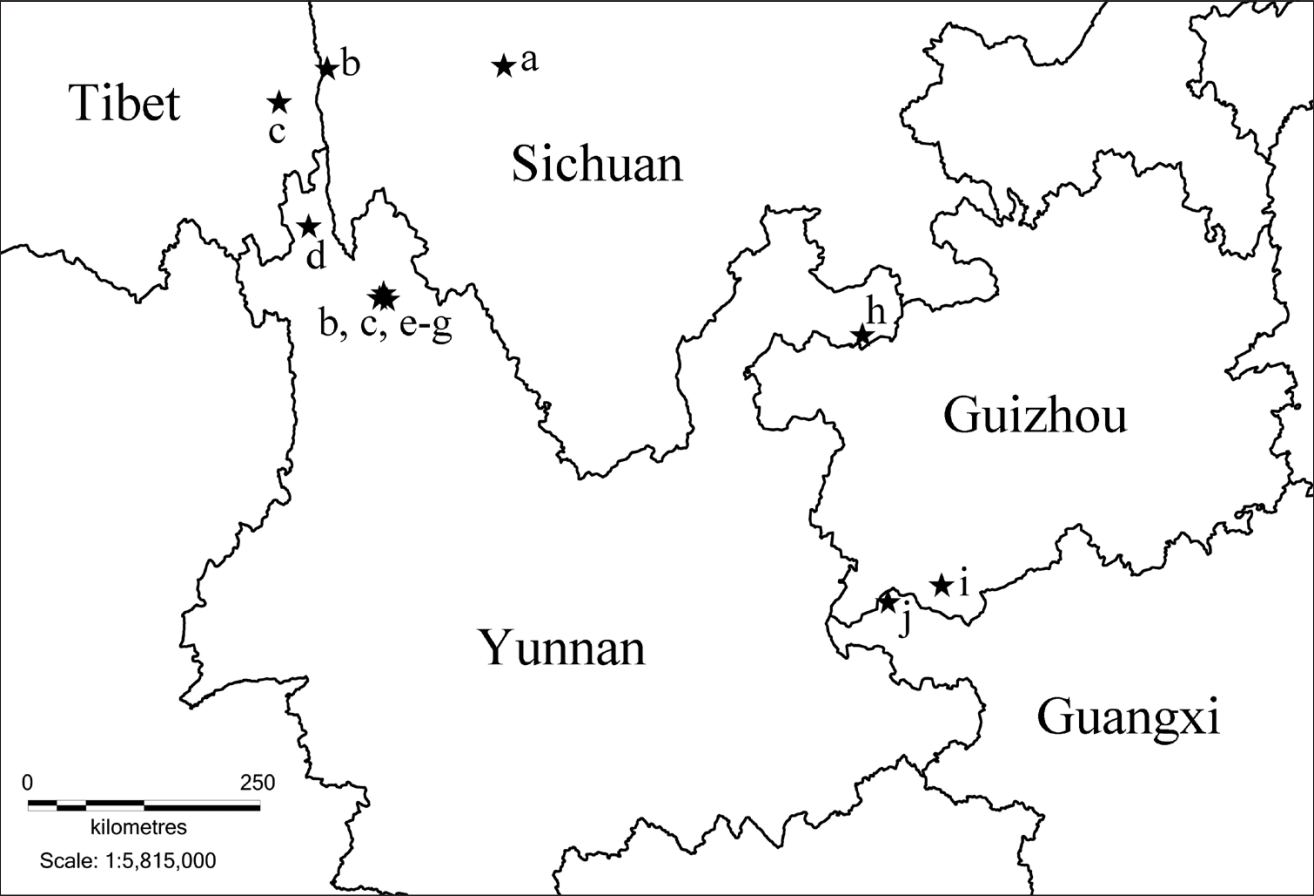
Distribution map of *Paracortina* in China. **a** *Paracortina voluta* **b** *Paracortina thallina* **c** *Paracortina viriosa* **d** *Paracortina serrata* **e** *Paracortina carinata* **f** *Paracortina leptoclada* **g** *Paracortina stimula* **h** *Paracortina chinensis* **i** *Paracortina zhangi* sp. n. **j** *Paracortina yinae* sp. n.

### Key to species of the genus *Paracortina*

**Table:** 

1	Gonopod prefemur with two processes covered with long macrosetae	**2**
–	Gonopod prefemur with only one process covered with long macrosetae	**8**
2	6+6 setae on pleurotergite 5	**3**
–	5+5 setae on pleurotergite 5	**4**
3	Head with a large median beak-shaped process located between antennae in males, 3+3 primary crests between poriferous crests	***Paracortina thallina***
–	Head without such a process, 5+5 primary crests between poriferous crests	***Paracortina stimula***
4	6+6 setae on pleurotergite 6 to penultimate	**5**
–	5+5 setae on pleurotergite 6 to penultimate	**6**
5	4+4 primary crests between poriferous crests; coxa 7 with two processes; gonopod prefemur with two processes clothed with dense long macrosetae apically	***Paracortina leptoclada***
–	3+3 primary crests between poriferous crests; coxa 7 without processes, gonopod prefemur’s nearby process smaller, with 8–9 strong macrosetae apically	***Paracortina voluta***
6	Coxa 7 with two processes; gonopod prefemur’s nearby process twisted, without setae	***Paracortina serrata***
–	Coxa 7 without process; gonopod prefemur’s nearby process apical with several apical setae	**7**
7	Collum with two large crests; gonopod telopodite’s terminal part trifid	***Paracortina viriosa***
–	Collum normal; gonopod telopodite’s terminal part bifid	***Paracortina carinata***
8	Body with 81–85 pleurotergites	***Paracortina multisegmentata***
–	Body with 50–65 pleurotergites	**9**
9	Gonopod coxa without any process	***Paracortina warreni***
–	Gonopod coxa with two processes	**10**
10	Gonopod coxa with a large, subtringular, ovoid anterior process (**a**) and a small tooth (**b**)	***Paracortina chinensis***
–	Gonopod coxa with a large, arch-shaped, anterior process (**a**) and a rather long and slender process (**b**) (Figs 17, 30)	**11**
11	Coxa 6 with a small, pointed, posterior process (**e**) (Fig. 30)	***Paracortina yinae* sp. n.**
–	Coxa 6 normal, without process	***Paracortina zhangi* sp. n.**

## Supplementary Material

XML Treatment for Paracortina zhangi

XML Treatment for Paracortina yinae
